# Notch (In)Sensitivity of Aluminum Matrix Syntactic Foams

**DOI:** 10.3390/ma12040574

**Published:** 2019-02-14

**Authors:** Attila Szlancsik, Bálint Katona, Dóra Károly, Imre Norbert Orbulov

**Affiliations:** 1 Department of Materials Science and Engineering, Faculty of Mechanical Engineering, Budapest University of Technology and Economics, Műegyetem rakpart 3., 1111 Budapest, Hungary; szlancsik@eik.bme.hu (A.S.); katona@eik.bme.hu (B.K.); kdora@eik.bme.hu (D.K.); 2 MTA–BME Lendület Composite Metal Foams Research Group, Műegyetem rakpart 3., 1111 Budapest, Hungary

**Keywords:** metal matrix composites, cellular materials, metallic foams, syntactic foams, mechanical characterization, fracture toughness, fracture surface, failure modes

## Abstract

Aluminum alloy (Al99.5 or AlSi12)-based metal matrix syntactic foams (MMSFs) were produced by pressure infiltration with ~65 vol % Globocer filler (33 wt % Al_2_O_3_, 48 wt % SiO_2_, 19 wt % Al_2_O_3_∙SiO_2_). The infiltrated blocks were machined by different geometry tools in order to produce notched samples. The samples were loaded in three-point bending, and the loading force values were recorded against the cross-head displacements and the crack opening displacements. To measure up the notch sensitivity and toughness of the MMSFs, the fracture energies and the fracture toughness values were determined. The results showed that the mentioned quantities are needed to describe the behavior of MMSFs. The fracture energies were shown to be notch-sensitive, while the fracture toughness values were dependent only on the matrix material and were insensitive to the notch geometry. The complex investigation of the fracture surfaces revealed strong bonding between the hollow spheres and the Al99.5 matrix due to a chemical reaction, while this bonding was found to be weaker in the case of the AlSi12 matrix. This difference resulted in completely different crack propagation modes in the case of the different matrices.

## 1. Introduction

Metal matrix syntactic foams (MMSFs) or composite metal foams (CMFs) are foams with high specific strength. In MMSFs the incorporated porosities—necessary for a foam structure—are ensured by low wall thickness hollow spheres. This structure leads to higher relative density (~0.5) foams exhibiting outstanding compressive properties compared at least to the “conventional” open and/or closed cell metallic foams. MMSFs can be produced from any kind of metal in theory, but Al alloys are the most common [1,2,3,4,5,6,7,8,9,10], but Mg [11,12,13,14,15,16,17], Fe [18,19,20,21,22,23,24,25], Ti [26,27,28,29], and even Zn [30,31,32,33]-based MMSFs can also be found in the literature. The filler material can also be various ranging from mixed oxide ceramics [15,34,35,36,37,38,39,40] to high purity and quality alumina [41,42,43,44] or silicon carbide [2,10,12]. Moreover, Taherishargh et al. have studied the application of cheaper expanded perlite as filler material [45,46,47,48,49,50,51,52,53,54,55]. Most of the above mentioned studies deals with the room temperature compressive properties of MMSFs, but efforts have been made to map their properties at cryogenic and elevated temperatures [33,56,57,58,59].

Recently, weight reduction and increasing loads on low-weight structures enhance the more extensive application of low absolute density foams having high specific strength. As a consequence, the mechanical and fracture properties as well as the failure mechanisms of metallic foams are becoming increasingly important. According to the authors, no comprehensive information is available up to now for MMSFs in this field. Only a few papers have been published on the toughness, fracture behavior, and even the crack propagation of “conventional” metallic foams.

In the simplest way, Chernousov and Chan defined the toughness as the absorbed mechanical energy (in Jcm^−3^ and its specific value, with respect to the density in Jg^−1^) during compression up to the appearance of the first crack [60]. The corresponding standard about the mechanical testing of cellular metals (ISO13314-2011 Mechanical testing of metals–Ductility testing–Compression test for porous and cellular metals [61]) does not contain any specification for the toughness of the material. It only defines the energy absorption and the energy absorption efficiency of the cellular materials. The approach of Chernousov and Chan is interesting and worth considering, as the main load type of foams is the compression; however, it is most often addressed as fracture energy, which can of course be interpreted as a specific kind of toughness as well.

McCullough et al. applied a more classic fracture mechanics approach and investigated the toughness of closed cell AlMg1Si0.6 and AlMg1Si10 foams, with relative density range of 0.1–0.4. Compact tension (CT) samples were tested based on the J-integral theory. The effect of the materials composition and the relative density on the toughness was evaluated and discussed in detail. Low Si content resulted in tougher foams, while the fracture toughness increased with the relative density. The authors also found that the pre-fatigue procedures suggested in the corresponding standards (ASTM E399 [62], ASTM E813 [63] (withdrawn) and ASTM E1152 [64] (withdrawn), and ASTM E1820 [65]) are negligible and the difference between the results of pre-fatigue and simply notched samples is indistinguishable. The foams exhibited increasing crack propagation resistance with crack growth, which was explained by the development of a certain crack bridging zone behind the crack tip. Another important finding of McCullough et al. was the notch insensitivity of the investigated closed cell “conventional” foams (tested on samples with a central, go-through hole). In both tensile and compressive load, the strength of the net cross section of the notched samples was equal to the un-notched strength [66].

Olurin et al. described the tensile and compressive deformation and the fracture properties of closed cell metallic foams in terms of their microstructures. Besides other mechanical properties, the notch sensitivity and the toughness of the foams were tested on double edge notched and CT samples, respectively. Tests on notched samples proved the notch-insensitivity of the investigated foams. During the toughness tests, a significant R-curve behavior was observed due to a bridging effect behind the actual crack tip, caused by the special microstructure of the foams [67].

Subsequently, the same research group studied the fatigue crack propagation. The investigated foams were in the 0.1–0.4 relative density range, and the ASTM E647 [68] standard was applied for the tests at 20 Hz. The authors found that linear elastic fracture mechanics can be used to describe the fatigue crack advance. The Paris–Erdogan relationship was applied to evaluate the results, and the exponent of the relationship proved to be quite high (m = 20–25). The high exponent led to the suggestion to keep the ΔK stress intensity range below the threshold (ΔK_th_) by proper design process for safety [69].

Motz et al. published two studies in the early 2000s. The first one dealt with ductile closed cell metallic foams in the aspects of fracture behavior and fracture toughness [70], while the second one discussed the fatigue crack propagation in cellular metals [71]. The densities of the investigated closed cell foams were in the 0.2–0.4 gcm^−3^ range. The tests were performed on different size CT samples (W = 50–300 mm). The pre-crack, which is required for the tests, was produced in different ways: by fatigue pre-cracking, by a razor blade polishing method (notch tip radius ~30 μm), and by a cut with the diamond wire saw (notch tip radius ~150 μm). The differences between the results of the three different notch tip preparations were indistinguishable, as was also observed by McCullough et al. [66,70]. To characterize the materials K_IC_ tests, J_IC_ tests and COD tests were performed. Due to the plasticity of the foams, the K_IC_ tests were not valid. The J_IC_ tests resulted in valid values, but the initiation toughness showed large scatter. The toughness was significantly influenced by the cell wall and edge thicknesses as well as by the inhomogeneities in the foams [70]. Later, Motz et al. performed a fatigue crack propagation test on the same foams and on a 316 L made hollow sphere structure (locally bonded stainless steel hollow spheres) with 0.3 gcm^−3^ density. The tests were performed at 20 Hz on CT samples (W of 50 and 140 mm). According to the test, again quite large Paris–Erdogan exponents (m = 6–12) were reported [71].

Combaz and Mortensen studied replicated Al foams, produced by the replication of the voids in a pack of NaCl particles with the relative density range of 0.10–0.24. The tests were performed on disk shape compact tension (DCT) samples. The measurements were evaluated according to the ASTM E1820 [65] standard. None of the tests resulted in valid K_IC_ values, so the data were evaluated for the R-curves and for the critical J-integral value for crack. During the fracture of the samples, a strong bridging effect behind the crack tip was observed [72]. Later their focus turned to the hole and notch sensitivity of replicated Al foams and found strong stress triaxiality in the ligament of notched samples [73].

Kashef et al. studied titanium alloy foams for medical application in the aspect of fracture toughness [74] and fatigue crack propagation [75]. The foams were made by powder metallurgy from 45 μm average size 99.9% purity titanium and an ammonium bicarbonate NH_4_HCO_3_ space holder (a purity of 99.0% and a size range of 500–800 μm). Two foams were produced with the relative densities of 0.3 and 0.4, respectively. CT samples were machined from the produced blocks according to the ASTM E1820 [65] standard and the samples were notched by a wire. The R-curves of the samples were measured. The authors reported that the titanium foams with higher relative density were tougher than the ones with lower relative density. Regarding the failure mechanism, the titanium CT samples had a plastic collapse in their ligament [74]. The authors later performed fatigue crack propagation tests on the higher relative density samples. Again, relatively high Paris–Erdogan exponents were reported (m ≈ 17) [75].

As is obvious from the above-mentioned papers, the community interested in metallic foams has invested some effort into the study of the notch sensitivity, crack initialization, and fatigue crack growth of various but “conventional” metallic foams, either open or closed cell. However, there are no results, nor have there even been any attempts, with respect to the notch sensitivity and toughness of MMSFs or CMFs. According to this, the main aims of this paper are to describe the notch sensitivity and to determine the fracture toughness of specific, aluminum alloy-based, ceramic hollow sphere-filled MMSFs.

## 2. Materials and Methods

Al99.5 or AlSi12 MMSFs were produced by inert gas pressure infiltration [76]. The chemical compositions of the matrices are listed in Table 1. As filler, mixed oxide ceramic hollow spheres (available under the trade name Globocer (GC), provided by Hollomet GmbH, Dresden, Germany) were applied at ~65 vol % [77,78]. The chemical composition of the hollow spheres was measured as 33 wt % Al_2_O_3_, 48 wt % SiO_2_, and 19 wt % Al_2_O_3_∙SiO_2_) [11,12]. The infiltration temperature was T_melting_ +50°C (710°C for Al99.5 and 625°C for AlSi12 matrices, respectively). The infiltration pressure and time were set to 400 kPa and 30 s, respectively.

**Figure 1:**
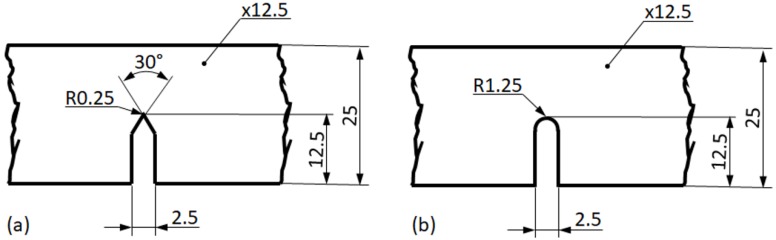
The investigated notch geometries: (**a**) sharp notch; (**b**) blunt notch.

Samples for three-point bending tests were machined from the infiltrated blocks (Figure 1). The width (W), the thickness (B), and the length (L) of the samples were W = 25 mm, B = 12.5 mm, and L = 105 mm, respectively. The span (S) of the three-point bending apparatus was S = 100 mm, while the diameter of the supporting and loading rods was 10 mm. In order to investigate the notch sensitivity of the produced MMSFs, the samples were manufactured with different notches (see Figure 1): (i) with a sharp, 12.5 mm long notch, identical to a fracture mechanics three-point bending (TPB) sample according to [62] (notch tip radius R = 0.25) and (ii) with a blunt, 12.5 mm long notch (notch tip radius R = 1.25 mm). All of the notches were straight and went through the whole thickness of the samples. The samples were designated according to their matrix material and notch type. For example, Al99.5-U-1 means the first Al99.5 matrix, GC-filled MMSF with a U notch.

Due to the findings of McCollough et al., no pre-fatigue cracks were initialized, because the difference between the results of pre-fatigue and simply notched samples was indistinguishable [66]. At least three samples were tested for each notch and material configuration (12 samples in sum). During the tests, the opening of the notch was followed by a double cantilever clip-in displacement gage. The cross head speed of the test was 1 mm/min. The tests were performed on an Instron 5965 testing machine (Instron, Norwood, MA, USA). During the tests, the load was recorded as a function of the notch opening. For the macroscopic observations of the fracture surfaces, an Olympus SZX-16 type microscope (Olympus Corporation, Tokyo, Japan) was used. The fracture surfaces were also scanned and mapped with a VHX 5000 microscope (Keyence, Osaka, Japan) in order to obtain 3D insight from the development of the fracture surface. The fracture surfaces were also investigated by scanning electron microscopy (SEM, Zeiss EVO MA10, Carl Zeiss AG, Oberkochen, Germany) extended with energy dispersive spectrometry (EDS, EDAX Z2, EDAX Inc., Mahwah, NJ, USA).

## 3. Results and Discussion

**Figure 2:**
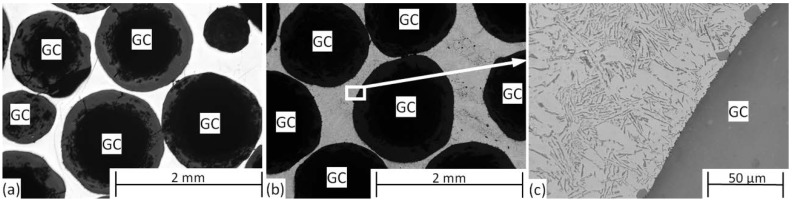
Micrographs of (**a**) Al99.5-based and (**b**) AlSi12-based metal matrix syntactic foams (MMSFs); (**c**) magnified part from subfigure (**b**).

Based on a short structural insight (to reveal the macroscopic and microscopic features of the materials), the results are summarized and discussed in this section according to two approaches, namely in terms of (i) toughness and (ii) fractographic features. Figure 2 represents the micrographs of Al99.5- and AlSi12-based MMSFs. The micrographs show almost perfect infiltration. During the production process, the molten alloy (lighter phase in the figures) could infiltrate even the narrowest gaps between the adjacent hollow ceramic spheres (labeled by “GC”). In the case of the AlSi12 matrix, the eutectic structure can be observed (Figure 2c).

**Figure 3:**
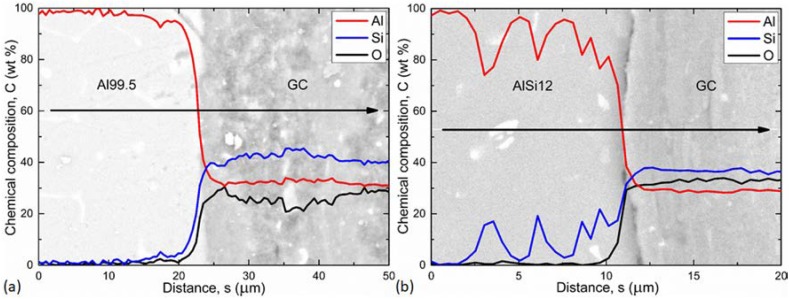
Line EDS element profiles of (**a**) Al99.5-based and (**b**) AlSi12-based MMSFs.

The structure of the MMSFs was further investigated by SEM. The aim of these measurements was to investigate the connection between the hollow spheres and the matrix material, which is crucial in the structural integrity point of view. The connection can be adhesive or cohesive. In the case of adhesive connection, the bonding is based only on the geometrical features of the hollow spheres’ surfaces. In the case of cohesive connection, a thin interface layer is formed during the production of the MMSFs, due to the possible chemical reactions between the molten metal and the ceramic hollow spheres (the molten Al reduces the SiO_2_ content of the hollow spheres to form Al_2_O_3_ and solve Si:4Al + 3SiO_2_ = 2Al_2_O_3_ + 3Si). In reality, the two phenomena are often overlapping and result in a complex connection. To investigate the interface layers, EDS measurement along lines perpendicular to the interface layer between a hollow sphere and the matrix material was performed (Figure 3).

**Figure 4:**
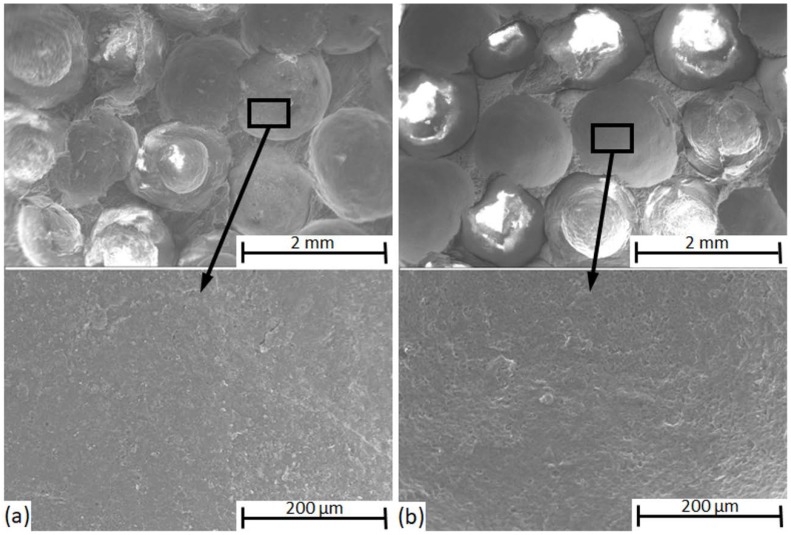
SEM micrographs of craters from the hollow spheres are removed during the fracture phenomenon: (**a**) Al99.5-based and (**b**) AlSi12-based MMSFs.

The line EDS profiles show the actual chemical composition (in wt %). The transition in the lines of the elements identify ~5.0 and ~2.5 μm thick interface layers in the case of Al99.5 and AlSi12 matrix, respectively (Figure 3). These interface layer thicknesses are quite low (especially in the case of the AlSi12 matrix, which was loaded by the ~0.3 μm uncertainty of the measurement due to the impact volume of the electron beam), so the chemical reaction between the matrix and the ceramic hollow spheres was constrained to the formation of an ideally thin interface layer in the case of the Al99.5 matrix. The large fluctuations in Figure 3b are due to the presence of Si lamellae in the eutectic AlSi12 matrix. The Si precipitations can also be observed in the SEM image of Figure 3b (light area along the investigation line). The Si lamellae sometimes remain “hidden” below the surface, but they can be tracked in the diagram, since the EDS technique investigates a larger volume of the material, not only the surface features. The qualitative difference between the two matrix materials is due to the relatively high Si content of the AlSi12 matrix (12 wt %), which hinders the diffusive chemical reactions, resulting in a weaker bonding between the spheres and the AlSi12 matrix. Besides the investigation of the cohesive connection, the SEM measurements were ideal for a closer look at the surface of the matrix material in sites from which the hollow spheres were removed during the fracture. Such SEM micrographs are represented in Figure 4.

In both subfigures, the smooth surface of the craters from which the ceramic hollow spheres were removed during the crack propagation and fracture can be clearly observed. The ratio of the adhesive bonding force within the whole bonding force between the constituents can be considered low; however, this is indirect evidence. The actual bonding force is hard to measure directly, but the qualitative investigation detailed above will be a good background for the investigation of the fracture surfaces (in Section 3.2) and will be beneficial to understand the crack propagation in the MMSFs.

### 3.1. Toughness

**Figure 5:**
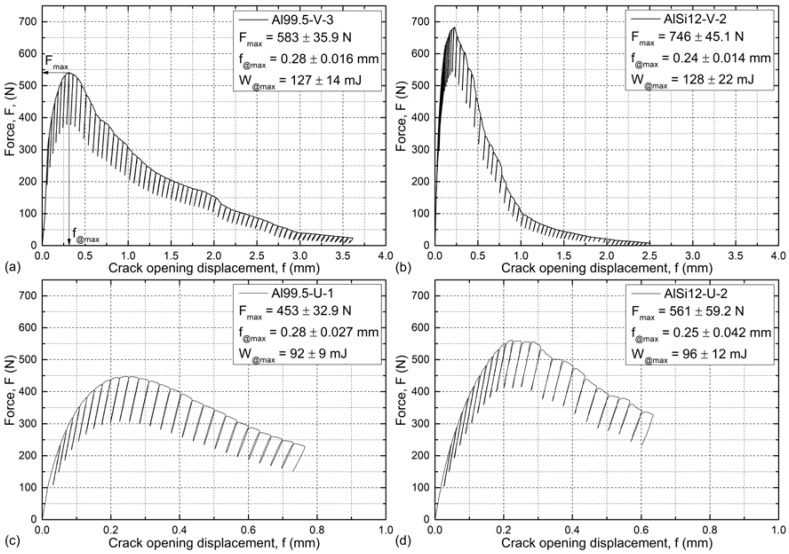
Typical force–crack opening displacement curves of (**a**) Al99.5-V, (**b**) AlSi12-V, (**c**) Al99.5-U, and (**d**) AlSi12-U MMSFs.

The typical load–crack opening curves for the investigated material and notch combinations with the unloading cycles are shown in Figure 5. In general, after a linear elastic part, a short range plastic deformation occurred and resulted in a maximal force. Up to this point, no pop-ins were detected. At the load maximum, a crack initialized at the notch tip and subsequently propagated, while the force decreased continuously. The force decrement was steeper in the case of the AlSi12 matrix due to the preferred crack propagation mode, detailed later in Section 3.2. The maximal force values and the corresponding crack opening displacements as well as the energy values up to the maximal fore (until the initialization of the first crack) are characteristic properties of the investigated MMSFs; they are listed in Figure 5.

The AlSi12 matrix MMSFs showed higher (~+25%) F_max_ values in all cases compared to the Al99.5 matrix foams. Due to the more brittle nature of the matrix, AlSi12 MMSFs showed higher scatter in their maximum forces. Similarly, the higher maximal forces were connected to lower (~−15%) crack opening displacements. In the notch tip geometry point of view, the notches with blunter tips resulted in lower maximal forces, while the corresponding crack opening displacements were geometry-independent and remained the same. The absorbed mechanical energies up to the appearance of the initial cracks (fracture energy, W_@max_) at the F_max_ values were calculated via numerically integrating the force–cross-head displacement curves (very similar to the F–f curves) up to the F_max_ force values (summarized in Table 2). These energies can be interpreted as toughness quantities since they show the energy required to break the samples. The fracture energy values were affected by the geometry of the notches only, so this method is not suitable to investigate the effect of the matrix material but yields notch-sensitive results. The samples with different matrix materials showed almost identical W_@max_ values. The effect of force increment and displacement decrement compensated each other; however, the higher scatters for the AlSi12 matrix were inherited to the fracture energy values as well. Regarding the notch geometries, unexpectedly, the blunter notches showed lower fracture energies, which can be explained by the higher probability of critical sites along the surface of the notch in the vicinity of the notch tip (refer to Section 3.2, on fractography) and, due to this, the higher probability of initializing the crack at lower force (and energy) values.

**Figure 6:**
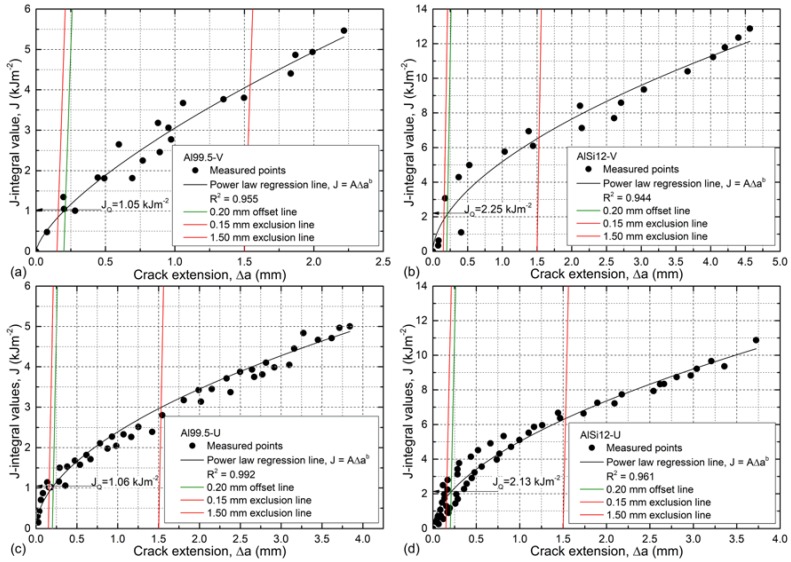
J–R curves of (**a**) Al99.5-V, (**b**) AlSi12-V, (**c**) Al99.5-U, and (**d**) AlSi12-U type MMSFs.

From a fracture mechanics point of view, all of the combinations show elastic-plastic behavior, and no K_IC_ values can be calculated by the 95% secant method, since the criterion F_max_/F_Q_ < 1.1 is clearly violated [65]. Therefore, the resistance curve approach was applied to evaluate the measurements. In this method, the force–crack opening displacement curves are transformed to a J-integral–crack extension curve (J–R curve, according to [65]). The J–R curves can be then evaluated for the questionable value of J-integral (J_Q_), which can be checked as to whether it is a critical value of J-integral and can be considered as dimension-independent fracture toughness (J_IC_) (in this case, J_Q_ is only valid for the investigated thickness of the sample). The J–R curves for the investigated configurations are plotted in Figure 6.

The J–R curves plot the agglomerated data of all samples within one MMSF type. Power law lines corresponding to the equation J = A · Δa^b^ were fitted on the measured points with R^2^ > 0.944 values. The fittings can be considered good. According to the standard, the 0.15 and 1.50 mm exclusion lines were constructed (red lines in Figure 6) as well as the 0.20 mm blunting line (green lines in Figure 6), which is a construction line that is key to finding the J_Q_ value as the intersection point with a fitted regression line (marked by labeled arrows in Figure 6). The slope of the blunting line was determined as 2σ_Y_, where σ_Y_ is the so-called effective yield strength (50 MPa for Al99.5-based and 115 MPa for AlSi12-based MMSFs respectively). The determined J_Q_ values are valid fracture toughness (J_IC_) values if both the original ligament thickness b_0_ (b_0_ = W − a_0_, where a_0_ is the length of the machined notch) and the thickness B are larger than 25 J_Q_/σ_Y_. These criteria were fulfilled in every case, so the J_Q_ values are valid fracture toughness (J_IC_) values. The obtained fracture toughness values are one magnitude higher than measured in the case of “conventional” foams [66,72]. Considering the effect of the matrix material and the notch geometry, the situation is contrary to the experienced in the fracture energy evaluation. The fracture toughness was affected by the matrix material only, so in this aspect the MMSFs were found to be notch-insensitive (Table 2). 

Due to this phenomenon, the judgement of the toughness of an MMSF structure is a complex decision and depends on the notch geometry (represented by W_@max_) and on the matrix material (through J_IC_). Among the investigated material and notch geometry combinations, the AlSi12-V type MMSFs performed better since they unite high fracture energy and high fracture toughness.

### 3.2. Fractography

**Figure 7:**
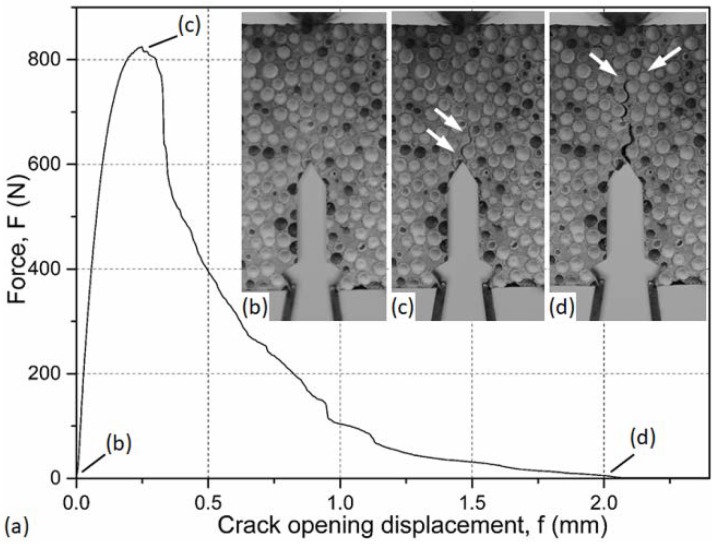
Fracture process of notched MMSFs (**a**) the force–crack opening displacement curve and the fracture surface of the AlSi12-V-1 sample; (**b**–**d**) crack propagation in the sample.

First, the fracture process during the loading of the samples was observed. Figure 7 shows the force–crack opening diagram for the AlSi12-V-1 sample and the corresponding pictures taken from the surfaces of the sample, showing the crack propagation and crack surface after the test. Starting from zero load, the specimen was unharmed and ready for the test (Figure 7b). As the increasing load reached its maximum a crack initialized in the notch tip (Figure 7c), the crack path ran along the interfaces between the hollow spheres and the matrix material (arrows in Figure 7c,d). In the case of the AlSi12 matrix, due to its relative brittleness (compared to Al99.5) normally an uncertainty appeared in the recorded load diagram (small amplitude waves just after the peak). As the crack propagated, the load gradually decreased (Figure 7a). In a point in the load–crack opening diagram, a sudden drop appeared and the crack branched into subcracks, as can be observed in Figure 7d, shown by the arrows. Finally, the sample broke into two parts.

**Figure 8:**
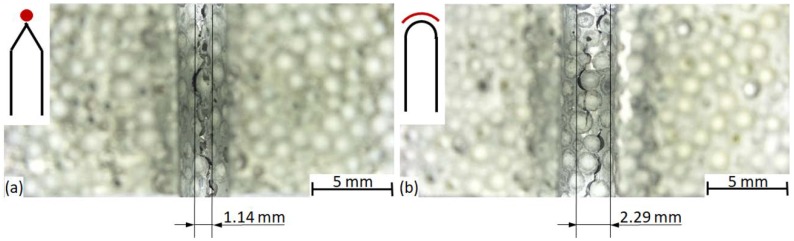
Initialized crack in the (**a**) AlSi12-V-3 and (**b**) Al99.5-U-3 sample.

As is mentioned in Section 3.1, the different notch geometries allowed different crack initializations, which is the reason behind the unexpectedly lower fracture energies in the case of blunter notches. The surface for the crack initialization is larger in the case of blunter U notches, since the stress concentration is lower and a similar stress distribution can be found on the perimeter of the notch tip, as is sketched in red at the top-left corners of the subfigures in Figure 8, representing the macrographs of the notch tips from the direction of the notch (referring to Figure 1, the viewpoint is from below).

**Figure 9:**
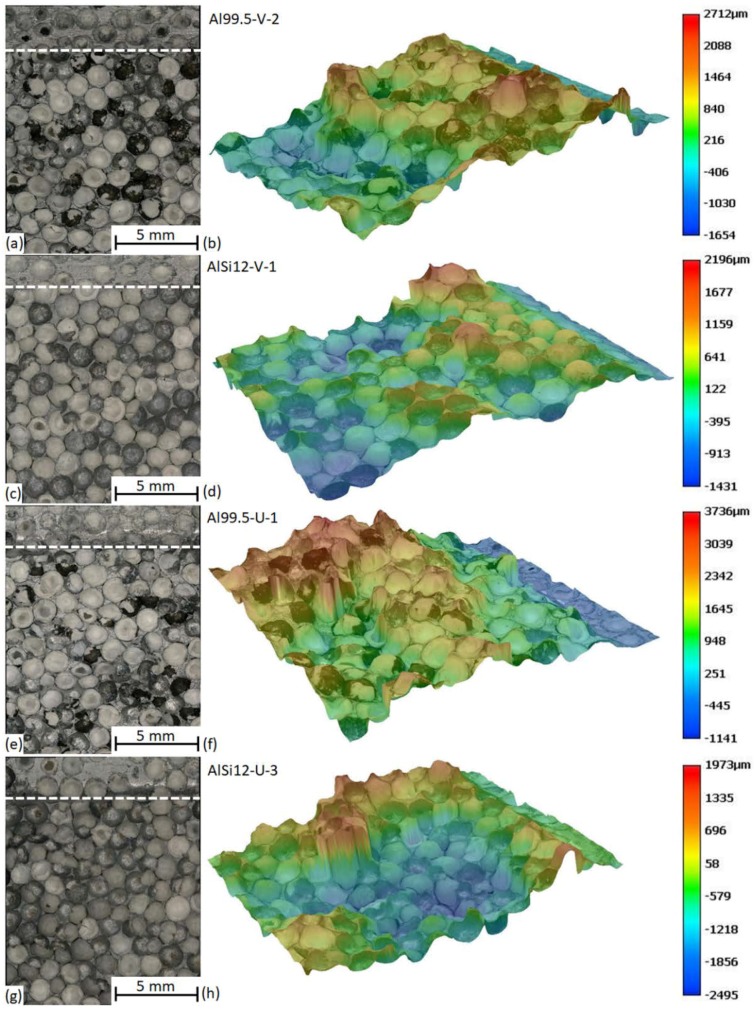
Typical fracture surfaces of MMSFs: (**a**,**b**) Al99.5-V-2, (**c**,**d**) AlSi12-V-1, (**e**,**f**) Al99.5-U-1, and (**g**,**h**) AlSi12-U-3 sample.

In the case of the V notch, all of the stress is concentrated at the very tip of the notch, forcing the crack to initialize at this site within a small volume (the width of the affected zone was found to be 1.12 ± 0.11 mm). Contrarily, in the case of the U notch, the size of the available crack initialization site (surface) is larger (a width of 2.25 ± 0.24 mm), so the possibility of having a critical site in this larger surface is higher; due to this, the crack can be initialized by lower fracture energy levels. The fracture surfaces were further analyzed through 3D macroscopic images (Figure 9).

Figure 9 represents the macrographs and their corresponding 3D surfaces. These macrographs allow one to map the preferred crack propagation sites, and they yield information on the bonding between the hollow spheres and the matrix material. A simple method is to count the broken and unbroken hollow spheres in the macrograph. The broken spheres indicate strong bonding to the matrix, since the bond was strong enough to hold the spheres tight and the crack broke them. The unbroken ones mean that the bonding was weaker, and due to this the hollow spheres remained in one sample-half. The counted values are listed in Table 3.

In the case of the Al99.5 matrix, the ratio of the broken spheres to the number of all spheres in the fracture surface was about 55–60%, while this ratio was ~15% in the case of the AlSi12 matrix, proving weaker bonding. However, the distance required for the crack to propagate was longer in the case of the AlSi12 matrix, resulting in a higher JIC value and a more quickly increasing R-curve, as is plotted in Figure 6. The 3D images show some deviation from the middle plane of the sample, but the deviation remained within ±2 mm at maximum, and the extreme values were typically far from the crack initialization site.

## 4. Conclusions

From the above detailed experiments, results, and discussion, aiming to map the fracture behavior and notch sensitivity of MMSFs, the following conclusions can be drawn:

- MMSFs showed elastic-plastic fracture behavior; therefore, besides the measurement of the fracture energies, the R-curve approach is suggested for investigating the toughness of MMSFs.
- The fracture energy up to the maximal force values and the critical value of the J-integral are both necessary to judge the toughness of the MMSFs. The W_@max_ values were sensitive to the notch geometry, while the J_IC_ values were affected only by the matrix material.
- In the case of the U notches, the probability of the presence of a critical site at the end of the notch was larger, so U-notched samples broke at lower W_@max_ values.
- The crack initialized at the crack tip propagated differently in the different matrix MMSFs, resulting different failure modes. In the case of the Al99.5 matrix, the cracks went through the hollow spheres, and this fact indicates a high bonding strength between the hollow spheres and the matrix material. In the case of the AlSi12 matrix, almost all of the hollow spheres were bypassed by the crack that propagated along the surfaces of the weakly bonded hollow spheres, resulting in longer propagation distance and higher R-curves.

## Figures and Tables

**Table 1:** Chemical composition of the matrices (in wt %).

Alloy	Al	Si	Fe	Cu	Mn	Mg	Zn	V	Ti
Al99.5	99.070	0.250	0.400	0.050	0.050	0.050	0.050	0.050	0.030
AlSi12	87.019	12.83	0.127	0.002	0.005	0.010	0.007	0.000	0.000

**Table 2:** Results of the toughness measurements.

MMSF Type	Fracture Energy, W_@max_ (mJ)	Fracture Toughness, J_IC_ (kJm^−2^)
	Notch-Sensitive	Notch-Insensitive
Al99.5-V	127 ± 14	1.05
**AlSi12-V**	**128 ± 22**	**2.25**
Al99.5-U	92 ± 9	1.06
AlSi12-U	96 ± 12	2.13

**Table 3:** Ratio of the broken hollow spheres on the fracture surface.

MMSF Type	Broken Spheres	All Spheres	Ratio (%)
Al99.5-V	52	87	59.8
AlSi12-V	15	98	15.3
Al99.5-U	49	89	55.1
AlSi12-U	14	96	14.6
